# Comparative Outcomes of Amoxicillin and Amoxicillin-Clavulanate in the Treatment of Acute Rhinosinusitis in Adults with Comorbidities: A Retrospective Study

**DOI:** 10.3390/antibiotics15080762

**Published:** 2026-08-07

**Authors:** Ran Yaacov Miron, Revital Perlov Gavze, Shiraz Vered, Shirley Shapiro Ben David, Ori Liran

**Affiliations:** 1 Division of Health, Maccabi Healthcare Services, 27 Hamered St, Tel Aviv 6812509, Israel; 2 Family Medicine Department, Gray’s Faculty of Medical & Health Sciences, Tel Aviv University, Tel Aviv 6997801, Israel; 3 Infectious Diseases Unit, Maccabi Healthcare Services, 27 Hamered St, Tel Aviv 6812509, Israel

**Keywords:** acute rhinosinusitis, sinusitis, amoxicillin, amoxicillin-clavulanate, antibiotics

## Abstract

**Background/Objectives**: The optimal antibiotic strategy for acute rhinosinusitis (ARS) in patients with comorbidities remains unclear. Guidelines often recommend amoxicillin-clavulanate (amx/clv) for high-risk patients, despite limited comparative evidence. This study compared ARS outcomes of patients with comorbidities receiving amoxicillin and amx/clv. **Methods**: This retrospective cohort study utilized electronic medical records from the Israeli community (2012–2023). Adults aged ≥18 years with ≥1 selected comorbidity and an ARS diagnosis were categorized according to the type of antibiotic prescribed. The primary outcome was treatment failure, defined as a follow-up ARS visit with an antibiotic prescription within 30 days of the initial visit. Secondary outcomes included ARS complications, emergency room referrals, chronic or subacute sinusitis diagnosis, and 90-day mortality rates. Adjusted odds ratios were estimated using multivariable logistic regression. **Results**: Of the 38,810 eligible patients, 4953 were prescribed amoxicillin, 7436 were prescribed amx/clv, and 26,421 were not prescribed antibiotics. Treatment failure rates were not significantly different when comparing amoxicillin to amx/clv or either antibiotic to no antibiotic therapy. Chronic or subacute sinusitis was more frequent with amx/clv than with amoxicillin alone. No significant intergroup differences were observed in terms of complications, emergency room referrals, or mortality rates. These findings remained consistent in a sensitivity analysis of filled prescriptions and when analyzing the outcomes of individual comorbidities separately. **Conclusions**: Among adults with specified comorbidities diagnosed with ARS, the rates of treatment failure, complications, and ER referrals did not differ significantly between amoxicillin and amx/clv, supporting narrow-spectrum therapy.

## 1. Introduction

Acute rhinosinusitis (ARS) is a common clinical condition that develops in the context of upper respiratory tract infections and affects 13.3% of women and 9% of men annually in the United States [1]. It is characterized by inflammation of the nasal cavity and paranasal sinuses, and commonly manifests as facial pain, nasal congestion, purulent discharge, headache, and occasionally fever. Complicated ARS is defined as ARS with clinically evident extension of inflammation outside the paranasal sinuses and nasal cavity, whereas uncomplicated ARS is restricted to the paranasal sinuses and nasal cavity [2]. Most ARS cases are caused by viral infections. Rhinovirus is detected in approximately 50% of patients with sinusitis. Other viral etiologies include influenza A and B, parainfluenza, respiratory syncytial virus, coronavirus, human metapneumovirus, and adenovirus. Only 0.5–2% of ARS episodes have a bacterial etiology [1,2,3]. The primary bacterial pathogens include Haemophilus influenzae, Streptococcus pneumoniae, and Moraxella catarrhalis [4]. Risk factors for ARS include acute nasopharyngitis, allergic rhinitis, acute bronchitis, chronic sinusitis, and chronic obstructive pulmonary disease (COPD) [5]. Risk factors for complicated ARS include smoking, anatomical abnormalities, allergic rhinitis, asthma, recent hospitalization, and immune compromise [6,7,8,9].

The diagnostic criteria for adult ARS, as outlined in the 2025 update of the American Academy of Otolaryngology–Head and Neck Surgery (AAO-HNS) guidelines, emphasize clinical presentation, symptom duration, and severity. Bacterial ARS is suspected when symptoms persist for 10 days or more, when symptoms worsen within 10 days of ARS presentation after initial improvement, or in patients with severe symptoms such as fever, unilateral facial pain, and purulent nasal discharge lasting 3–4 days. These indications have a limited pretest probability (approximately 60% in some studies) [1,2,10]. Imaging is recommended for suspected complications of sinusitis, such as extra-sinus involvement of orbital, intracranial or soft tissue, a history of facial trauma or surgery, or when an alternative diagnosis such as malignancy is suspected [2]. Current guidelines reinforce a cautious approach to antibiotic use, encouraging shared decision-making and delayed prescription of antibiotics for up to 5 days after diagnosis. This can include prescribing antibiotics and informing the patient to withhold the purchase of the drug pending worsening symptoms or withholding prescription altogether while assessing for clinical improvement [2]. Due to the clinical nature of the diagnosis of bacterial ARS, distinguishing viral from bacterial ARS is challenging and depends on the patient’s description of symptoms as well as the physician’s physical examination. This can potentially lead to overprescription of antibiotics [11].

According to the Infectious Diseases Society of America (IDSA) and AAO-HNS guidelines, amx/clv is recommended for patients at high risk of infection with β-lactamase–producing bacteria or those with risk factors for penicillin-nonsusceptible Streptococcus pneumoniae, such as age > 65 years, recent hospitalization, antibiotic use within one month prior to ARS, severe infection, or immunocompromised status [2,10].

Choosing amx/clv over amoxicillin for bacterial ARS is also influenced by bacterial resistance patterns in specific geographical areas. For instance, β-lactamase-producing H. influenzae has a prevalence of 27% in the United States, and more specifically, penicillin-resistant S pneumoniae has a prevalence of approximately 41% in the southeast United States, which may influence antibiotic selection [2,12].

Although large cohort studies have demonstrated the equal effectiveness of amoxicillin and amoxicillin/clavulanate (amx/clv) in adults with ARS, data comparing these antibiotics in patients with specific comorbidities are limited. Previous studies have defined comorbidity as either immunodeficiency or a selection of chronic conditions, but did not analyze each specific condition separately [13,14]. Broader-spectrum antibiotics, such as amx/clv, contribute to antimicrobial resistance and increase the risk of adverse effects [15,16].

This study aimed to compare the outcomes of amoxicillin and amx/clv in the treatment of ARS in adults with specific comorbidities.

## 2. Results

### 2.1. Study Population

**Figure 1:**
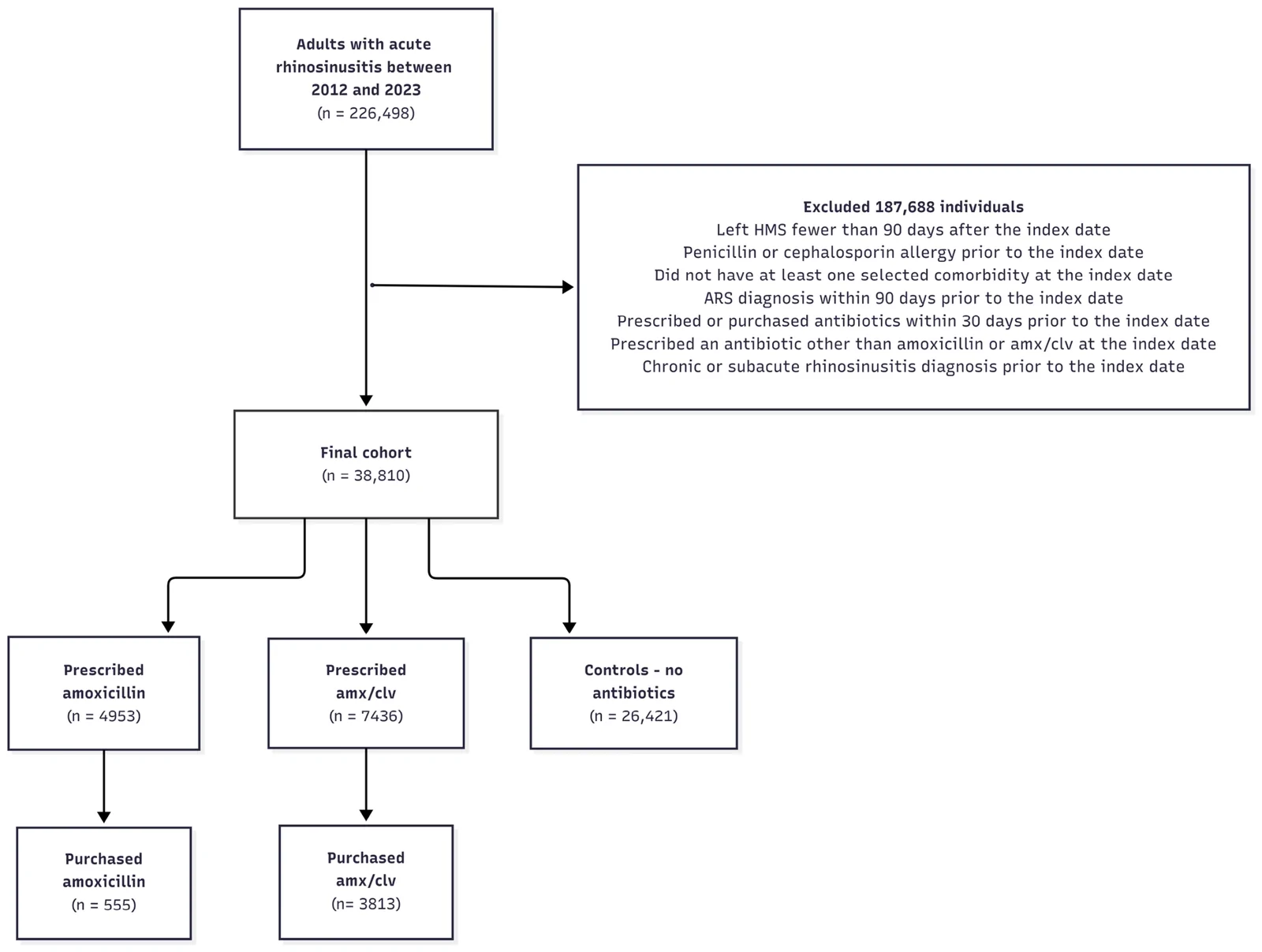
Study population flowchart.

Of the 38,810 eligible patients, 4953 were prescribed amoxicillin, 7436 were prescribed amx/clv, and 26,421 were not prescribed antibiotics [Figure 1]. Patient characteristics and comorbidities according to the prescription group are presented in Table 1 and Table 2.

### 2.2. Treatment Failure

Treatment failure occurred in 3.6% and 3.5% of the amoxicillin and amx/clv groups, respectively. There was no significant difference between groups (adjusted OR 0.99 [95% CI 0.81–1.21]) [Table 3]. Compared with the control group (3.8%), neither antibiotic group was associated with a significantly different failure rate (amoxicillin: adjusted OR 0.93, 95% CI 0.79–1.09; amx/clv: adjusted OR 0.93, 95% CI 0.81–1.07) [Table 4].

### 2.3. Chronic or Subacute Sinusitis

Diagnosis of chronic or subacute sinusitis within 90 days of the index date occurred in 0.7% and 1.9% of the amoxicillin and amx/clv groups, respectively. Chronic or subacute sinusitis diagnosis rates were significantly higher in the amx/clv group compared to the amoxicillin group (adjusted OR 2.62, 95% CI 1.81–3.80, NNH 83) [Table 3]. Compared with the control group (1.5%), the amoxicillin group had significantly lower rates (adjusted OR 0.49, 95% CI 0.34–0.68, NNT 125), and the amx/clv group had significantly higher rates (adjusted OR 1.27, 95% CI 1.05–1.54, NNH 250) [Table 4].

### 2.4. Complications

Sinusitis-related complication rates within 90 days following the index date were 1.2% and 0.9% in the amoxicillin and amx/clv groups, respectively, with no significant difference between groups (adjusted OR 0.73, 95% CI 0.51–1.05) [Table 3]. The complication rate in the control group was 1.1%, and neither antibiotic group demonstrated significantly different complication rates compared to the control group (amoxicillin: adjusted OR 1.08, 95% CI 0.81–1.44; amx/clv: adjusted OR 0.84, 95% CI 0.64–1.09) [Table 4].

### 2.5. Emergency Room Referrals

Emergency room referral rates due to ARS or ARS complications were 1.2% and 0.9% in the amoxicillin and amx/clv groups, respectively, with no significant difference between groups (adjusted OR 0.73, 95% CI 0.51–1.05) [Table 3]. Emergency room referral rate due to ARS or ARS complications in the control group was 1.1%, and there was no significant difference in emergency room referral rates between the control and amoxicillin groups (adjusted OR 1.07, 95% CI 0.81–1.43) and between the control and amx/clv groups (adjusted OR 0.83, 95% CI 0.64–1.08) [Table 4].

### 2.6. Mortality

Mortality within 90 days of the index date was rare in all groups. There were no deaths in the amoxicillin group, one death in the amx/clv group, and two deaths in the control group.

### 2.7. Sensitivity Analysis

A sensitivity analysis was conducted among patients who filled their antibiotic prescriptions, including 555 in the amoxicillin group and 3813 in the amx/clv group [Table 5]. Treatment failure occurred in 4.9% and 4.7% of the amoxicillin and amx/clv groups, respectively (adjusted OR 0.99 [95% CI 0.66–1.52]). Diagnosis of chronic or subacute sinusitis within 90 days following the index date occurred in 0.5% and 2.5% of the amoxicillin and amx/clv groups, respectively (adjusted OR 4.62 [95% CI 1.45–14.69 NNH 53]). Complication rates and emergency room referral rates were identical, 1.1% in the amoxicillin group and 1.2% in amx/clv group, with no significant difference between groups (adjusted OR 1.05, 95% CI 0.44–2.52). Mortality remained rare, with no deaths in the amoxicillin group and one death in the amx/clv group.

## 3. Discussion

Although Guidelines for ARS recommend amx/clv for patients at higher risk of amoxicillin-resistant organisms [2,10], this large retrospective cohort study, including 38,810 adults with different comorbidities treated for ARS, analyzed using both an intention-to-treat approach based on the antibiotic prescribed and a as-treated approach based on actual prescription fulfillment, found no significant differences in treatment failure, ARS complications, emergency room referrals due to ARS, or mortality between patients prescribed amoxicillin, amx/clv, or no antibiotics.

These real-world results are consistent with those of previous comparative effectiveness studies. In a U.S. Veterans Affairs cohort of nearly 90,000 adults, no significant difference was found in sinusitis-related return visits or infectious complications between amoxicillin and amx/clv [13]. The authors noted that their findings were limited to uncomplicated cases, suggesting that the study’s impact on patients with comorbidities was not specifically addressed. Another large-scale observational study of adults with ARS treated with amx/clv or amoxicillin found no difference in treatment failure rates [14]. Although subgroup analyses were performed for age, sex, and immunocompromised status, no subgroup analysis was performed for other comorbidities. In contrast, our study specifically focused on a population with chronic comorbidities, providing evidence that broader empirical antibiotic choices may not improve the outcomes of patients with these conditions.

Several factors may explain the treatment failure rates observed in our study. First, alternative antibiotics may have been initiated prematurely before the primary regimen achieved therapeutic efficacy. Second, switching in the amoxicillin-clavulanate group could be driven by its higher historical adverse event rate. Conversely, lower adherence in the amoxicillin cohort may stem from its more demanding three-times-daily dosing schedule compared to the twice-daily regimen of amoxicillin-clavulanate. Finally, lack of clinical improvement may reflect either amoxicillin failure against beta-lactamase-producing pathogens or inadvertent treatment of self-limiting viral acute rhinosinusitis [2,15].

Guidelines for ARS recommend high-dose amx/clv in patients at a higher risk of treatment failure due to suspected resistant organisms [2,10]. Our study did not differentiate between different amoxicillin and amx/clv dosing regimens. However, Randomized trials evaluating different amx/clv dosing strategies did not demonstrate an advantage of higher dosing in the general adult population [17,18]. A future study comparing different dosing strategies in adults with specific comorbidities may clarify the need for increased dosing of either antibiotic in this population.

A previous randomized trial found that amx/clv did not significantly hasten symptom resolution compared to placebo and was associated with more adverse effects [19]. A meta-analysis of randomized controlled trials concluded that although antibiotics shorten ARS duration compared to placebo, they confer only a small therapeutic benefit and are associated with a higher risk of adverse effects [20]. These studies often excluded individuals with comorbidities or did not stratify outcomes by comorbidity status, limiting their direct applicability to populations with comorbidities. Our study demonstrated no significant differences in rates of treatment failure, ARS complications, or emergency room referral rates when comparing either amoxicillin or amoxicillin-clavulanate to no antibiotic treatment in adults with comorbidities and ARS.

The increased incidence of chronic and subacute rhinosinusitis observed in the amx/clv group likely reflects residual confounding by indication. Clinicians may have preferentially prescribed broader-spectrum therapy to patients presenting with higher baseline disease severity or complex clinical features. Because baseline severity was unavailable in the database, these individuals, who are inherently at a higher risk for prolonged or recurrent disease progression, could not be fully adjusted for. Notably, no prior studies have demonstrated a causal association between amoxicillin-clavulanate exposure and the development of chronic or subacute sinusitis, further supporting a methodological rather than a biological explanation for this finding [20].

### Strengths and Limitations

This study had several strengths, including a large and representative cohort, inclusion of patients with a wide range of comorbidities, and access to longitudinal data from a comprehensive community healthcare database. The use of multivariable logistic regression, sensitivity analyses, and interaction analyses enhanced the robustness of our findings. However, this study had several limitations. As this was a retrospective observational study, we could not establish causality, and residual confounding may persist despite statistical adjustment. Underlying immunodeficiency was excluded from interaction and subgroup analyses. In our database, this variable represents a broad aggregate of heterogeneous codes that do not directly align with standard clinical guideline definitions (such as those from the AAO-HNS). Future dedicated studies are needed to disaggregate this composite entity and evaluate outcomes across specific immunocompromised subpopulations. The definition of treatment failure chosen in our study is a surrogate for actual failure and may have been contributed to by additional factors other than different antibiotics. Comparisons of different lengths and doses of antibiotic therapy were not performed in this study. A future study is planned to evaluate the outcomes of different antibiotic therapy regimens in patients with comorbidities and ARS. Although a sensitivity analysis was performed among patients who filled their prescriptions, verifying medication ingestion was not possible in this study. Prescriptions from primary care physicians and ENT specialists were analyzed together without stratification by physician specialty. Because patient complexity and management practices may differ between primary and specialty care settings, future research evaluating outcomes stratified by prescriber specialty is warranted. We were limited by the lack of clinical details, such as symptom severity and physical examination results. Because bacteriological studies for sinusitis are not routinely performed for uncomplicated sinusitis in the community setting, we did not have access to microbiological data, including nasal cultures and resistance patterns in this study. Therefore, differentiating between bacterial and viral ARS using clinical or bacteriological data was not possible. Antibiotic selection may have been biased by baseline disease severity, with more severe cases potentially receiving broader spectrum therapy. It is possible that analysis of the results was confounded by cases of viral ARS who received antibiotics, and vice versa. Hospital data, such as diagnoses at emergency room admission, ward admission diagnoses, or cause of death, were limited. Data on the use of decongestants, which may influence prognosis, were not available because of variability in formulations and frequent over-the-counter dispensing. The low event rate for mortality and certain complications may have limited our ability to detect significant differences in rare adverse outcomes. Because data collection spanned an 11-year period (2012–2023), temporal shifts in antimicrobial stewardship policies and clinical guidelines may have influenced treatment selection. Although our study prioritized comparative effectiveness over longitudinal trends, these evolving clinical thresholds represent a potential source of unmeasured variation.

## 4. Materials and Methods

### 4.1. Study Design and Setting

This retrospective cohort study used anonymized data from the Maccabi Healthcare Services (MHS) database in Israel. The MHS is a nationwide health maintenance organization (HMO) that serves over 2.7 million individuals. The study period was from January 2012 to September 2023. This study focused on adults diagnosed with ARS who had pre-existing comorbidities and received treatment at community clinics in Israel.

### 4.2. Eligibility Criteria and Cohort Selection

Eligible patients were aged ≥18 years and had a new diagnosis of ARS between 2012 and 2023. The first qualifying ARS diagnosis during the study period was defined as the index date of the study. Prescriptions issued by primary care physicians and ear, nose, and throat (ENT) specialists were analyzed as a single cohort. Study participants were chosen based on their increased potential for complicated ARS or pre-existing conditions for which the guidelines recommend amx/clv [2]. Patient characteristics were identified using MHS registries and ICD9 codes. Included patients had at least one of the following comorbidities at the time of the index date: diabetes, hypertension, allergic rhinitis, known septal deviations, age > 65 years, recent hospitalization (30 days prior to the index date), smoking history, asthma, COPD, chronic heart failure, chronic kidney disease, and chronic hepatic failure. Immunodeficiency was not included in the comorbidities selected for the interaction and subsequent subgroup analysis. This decision stemmed from it being a composite of many different diagnoses and registries selected by the HMS, and not necessarily equal to immunodeficiency status as defined by the AAO sinusitis guidelines. Patients were excluded if they purchased any antibiotic 30 days prior to the index date, suffered from an allergy to penicillin or cephalosporins prior to the index date regardless of severity, received a prescription for antibiotics other than amoxicillin or amx/clv on the index date, had chronic or subacute sinusitis diagnosis prior to the index date, or withdrew from the MHS within 90 days following the index date for reasons other than death.

### 4.3. Exposure Groups

Patients were categorized into three groups based on the prescription received on the index date: (1) amoxicillin, (2) amx/clv, and (3) no antibiotic treatment. Antibiotic prescriptions were identified using electronic health records. A sensitivity analysis was performed for patients who filled their prescriptions.

### 4.4. Outcomes

The primary outcome was treatment failure, defined as a follow-up visit with ARS diagnosis and a new antibiotic prescription within 30 days of the index date. ARS diagnosis was performed clinically by a family physician or an otolaryngologist using relevant medical history and physical examination. Our study did not require imaging or laboratory tests to confirm the diagnosis, as they are not required for bacterial ARS diagnosis and are generally indicated only when ARS complications are suspected. Secondary outcomes included diagnosis by a family physician or otolaryngologist of chronic sinusitis (sinusitis symptoms lasting more than 12 weeks) or subacute sinusitis (sinusitis symptoms lasting 4–12 weeks) according to history and physical examination within 90 days following the index date, emergency room referral due to ARS or sinusitis-related complications within 90 days following the index date, sinusitis-related complications within 90 days following the index date, and 90-day mortality following the index date. Sinusitis-related complications included orbital or periorbital cellulitis, meningitis, osteomyelitis, sinus vein thrombosis, and intracranial abscesses.

### 4.5. Data Sources and Definitions

All data, including diagnoses, prescriptions, registries, and demographic information, were extracted from a centralized electronic medical record system. Comorbidities were defined using standardized MHS disease registries and ICD-9 codes. Socioeconomic status (SES) was determined using the Central Bureau of Statistics (CBS) socioeconomic index of geographical statistical areas, a composite measure derived from demographic characteristics, education, standard of living, employment, and income indicators based on national administrative data. The index was calculated as a continuous score and reported in deciles ranging from 1 (lowest SES) to 10 (highest SES), with higher deciles indicating higher SES. Population sectors (Jewish, Arab, and ultra-Orthodox Jews) were categorized according to CBS definitions [21].

### 4.6. Statistical Analysis

Descriptive statistics were used to summarize demographic and clinical characteristics. Group comparisons were conducted using chi-square tests for categorical variables and t-tests or Mann–Whitney U tests for continuous variables, as appropriate. In addition to *p*-values, standardized mean differences (SMDs) were used to quantify the magnitude of differences between groups. When the three groups were compared, a summary SMD was reported for each variable, defined as the maximum absolute pairwise SMD across the three pairwise group comparisons. An SMD < 0.10 was considered indicative of a negligible imbalance. Multivariable logistic regression was used to assess the association between antibiotic treatment and binary outcomes. All covariates were entered into the model simultaneously, with no variable selection procedures applied. Absolute risk differences (ARDs) with 95% confidence intervals were calculated along with odds ratios. Depending on the direction and statistical significance of the effect, the Number Needed to Treat (NNT) or Number Needed to Harm (NNH) was reported for statistically significant outcomes. Adjusted odds ratios (aORs) and 95% confidence intervals (CIs) were reported for the results. Interaction analyses were conducted to assess whether the association between treatment and outcomes varied according to comorbidity status. Interaction terms between the treatment groups and each comorbidity were included in the regression models to evaluate potential effect modifications. No statistical adjustments were made for multiple comparisons. A sensitivity analysis was performed to assess the outcomes of patients who received and purchased antibiotics. Notably, baseline disease severity at presentation was unavailable, representing a potential unmeasured confounder in initial antibiotic selection. Statistical significance was defined as a two-sided *p*-value of <0.05. All analyses were performed using SPSS version 29.

### 4.7. Ethics

This study was approved by the Maccabi Institutional Review Board (MHS-0055-24). All data were anonymized before analysis. The requirement for informed consent was waived owing to the retrospective and non-interventional nature of the study.

## 5. Conclusions

Among adults with comorbidities such as diabetes, hypertension, allergic rhinitis, known septal deviations, age > 65 years, recent hospitalization (30 days prior to ARS), smoking history, asthma, COPD, chronic heart failure, chronic kidney disease, and chronic hepatic failure, diagnosed with ARS in a community setting, there were no significant differences between amoxicillin and amx/clv in terms of treatment failure, complications, or emergency room referral rates. Our findings support the narrow-spectrum use of antibiotics in this specific population treated for ARS.

## Figures and Tables

**Table 1:** Characteristics and demographic parameters of the study participants according to prescription: control, amoxicillin, and amoxicillin clavulanate groups.

Population Parameter	Control Group N = 26,421 N (%)	Amoxicillin Group N = 4953 N (%)	Amoxicillin Clavulanate N = 7436 N (%)	*p*-Value	Approximate Overall Standardized Mean Differences
Age at index date, mean ± std	42.10 ± 17.2	42.4 ± 16.8	41.7±16.6	0.061	0.04
Age at index date (Age ≥ 65)	3695 (14.0)	657 (13.3)	886 (11.9)	<0.001	0.06
Sex (male)	11,245 (42.6)	1993 (40.2)	3595 (48.3)	<0.001	0.16
Socioeconomic status					
1–4 (Low)	5088 (19.3)	807 (16.3)	1643 (22.1)	<0.001	0.15
5–7 (Med)	12,093 (45.8)	2299 (46.42)	3499 (47.1)	<0.001	0.03
8–10 (High)	9201 (34.82)	1842 (37.2)	2286 (30.74)	<0.001	0.14
Ethnicity (Jews)	24,978 (94.5)	4823 (97.4)	6892 (92.7)	<0.001	0.2
Smoking history	5664 (21.44)	777 (15.7)	1754 (23.6)	<0.001	0.19
allergic rhinitis	11,456 (43.36)	2215 (44.7)	3057 (41.1)	<0.001	0.07
known septal deviations	3426 (12.97)	571 (11.5)	949 (12.8)	0.021	0.04
Hospitalization in the past 30 days	362 (1.4)	55 (1.1)	112 (1.5)	0.174	0.04
Asthma	7449 (28.19)	1360 (27.5)	1999 (26.9)	0.067	0.03
Chronic hepatic failure	591 (2.2)	56 (1.1)	170 (2.3)	<0.001	0.1
Chronic heart failure	288 (1.1)	48 (1.0)	80 (1.1)	0.75	0.01
Diabetes	2555 (9.7)	523 (10.6)	819 (11.0)	0.001	0.04
Hypertension	5449 (20.6)	1237 (24.97)	1673 (22.5)	<0.001	0.1
Chronic kidney disease	3613 (13.7)	873 (17.6)	1051 (14.1)	<0.001	0.11
COPD	429 (1.62)	81 (1.6)	138 (1.9)	0.378	0.02
Immunodeficiency	932 (3.5)	139 (2.8)	240 (3.2)	0.026	0.04

**Table 2:** Characteristics and demographic parameters of the study participants according to prescription: amoxicillin and amoxicillin clavulanate groups.

Population Parameter	Amoxicillin Group N = 4953 N (%)	Amoxicillin Clavulanate N = 7436 N (%)	*p*-Value	Standardized Mean Differences
Age at index date, mean ± std	42.4 ± 16.8	41.7 ± 16.6	0.016	0.04
Age at index date (Age ≥ 65)	657 (13.3)	886 (11.9)	0.026	0.04
Sex (male)	1993 (40.2)	3595 (48.3)	<0.001	0.16
Socioeconomic status				
1–4 (Low)	807 (16.3)	1643 (22.1)	<0.001	0.15
5–7 (Med)	2299 (46.5)	3499 (47.1)	<0.001	0.01
8–10 (High)	1842 (37.2)	2286 (30.8)	<0.001	0.14
Ethnicity (Jews)	4823 (97.4)	6892 (92.7)	<0.001	0.2
Smoking history	777 (15.7)	1754 (23.6)	<0.001	0.2
allergic rhinitis	2215 (44.7)	3057 (41.1)	<0.001	0.07
known septal deviations	571 (11.5)	949 (12.8)	0.04	0.04
Hospitalization in the past 30 days	55 (1.1)	112 (1.5)	0.068	0.03
Asthma	1360 (27.5)	1999 (26.9)	0.483	0.01
Chronic hepatic failure	56 (1.1)	170 (2.3)	<0.001	0.09
Chronic heart failure	48 (1.0)	80 (1.1)	0.565	0.01
Diabetes	523 (10.6)	819 (11.0)	0.425	0.01
Hypertension	1237 (24.97)	1673 (22.5)	0.001	0.05
Chronic kidney disease	873 (17.6)	1051 (14.1)	<0.001	0.1
COPD	81 (1.6))	138 (1.9)	0.362	0.02
Immunodeficiency	139 (2.8)	240 (3.2)	0.182	0.03

**Table 3:** Outcome comparison of amoxicillin and amoxicillin clavulanate groups.

Outcome	Amoxicillin Group N = 4953 N (%)	Amoxicillin Clavulanate N = 7436 N (%)	Crude OR [95% CI], ARD	Adjusted OR [95% CI], ARD
Treatment failure—follow-up visits up to 30 days after the index date with ARS diagnosis and any antibiotic prescription	178 (3.6)	258 (3.5)	0.96 [0.79–1.17], −0.001	0.99 [0.81–1.21], −0.0008
Chronic or Subacute rhinosinusitis diagnosis up to 90 days following the index date	36 (0.7)	144 (1.9)	2.7 [1.87–3.89], 0.012	2.62 [1.81–3.80], 0.012
Rhinosinusitis complication—A diagnosis of a complication of ARS within 90 days of the index date. Complications include orbital or periorbital cellulitis, osteomyelitis, meningitis, sinus vein thrombosis, septic cavernous sinus thrombosis, subperiosteal abscess, and intracranial abscess	57 (1.2)	69 (0.9)	0.80 [0.57–1.15], −0.002	0.73 [0.51–1.05], −0.003
Emergency room referral—A referral given on a visit with ARS diagnosis or one of the above complications within 90 days of the index date	57 (1.2)	69 (0.9)	0.80 [0.57–1.15], −0.002	0.73 [0.51–1.05], −0.003

**Table 4:** Outcome comparison of amoxicillin and amoxicillin clavulanate vs. control groups.

Outcome	Control Group N = 26,421 N (%)	Amoxicillin vs. Control			Amoxicillin Clavulanate vs. Control		
		Amoxicillin Group N = 4953 N (%)	Crude OR [95% CI], ARD	Adjusted OR [95% CI], ARD	Amoxicillin Clavulanate Group N = 7436 N (%)	Crude OR [95% CI], ARD	Adjusted OR [95% CI], ARD
Treatment failure—follow-up visits up to 30 days after the index date with ARS diagnosis and any antibiotic prescription	999 (3.8)	178 (3.6)	0.95 [0.81–1.11], −0.002	0.93 [0.79–1.09], −0.003	258 (3.5)	0.92 [0.80–1.05], −0.003	0.93 [0.81–1.07], −0.003
Chronic or Subacute rhinosinusitis diagnosis up to 90 days following the index date	402 (1.5)	36 (0.7)	0.47 [0.34–0.67], −0.008	0.49 [0.34–0.68], −0.008	144 (1.9)	1.28 [1.06–1.55], 0.004	1.27 [1.05–1.54], 0.004
Rhinosinusitis complication—A diagnosis of a complication of ARS within 90 days of the index date. Complications include orbital or periorbital cellulitis, osteomyelitis, meningitis, sinus vein thrombosis, septic cavernous sinus thrombosis, subperiosteal abscess, and intracranial abscess	285 (1.1)	57 (1.2)	1.07 [0.80–1.42], 0.0008	1.08 [0.81–1.44], 0.0008	69 (0.9)	0.86 [0.66–1.12], −0.002	0.84 [0.64–1.09], −0.002
Emergency room referral—A referral given on a visit with ARS diagnosis or one of the above complications within 90 days of the index date	286 (1.1)	57 (1.2)	1.06 [0.80–1.42], 0.0007	1.07 [0.81–1.43], 0.0008	69 (0.9)	0.86 [0.66–1.12], −0.002	0.83 [0.64–1.08], −0.002

**Table 5:** Sensitivity analysis comparing amoxicillin and amoxicillin clavulanate groups in patients who filled their prescriptions.

Outcome	Amoxicillin Group N = 555 N (%)	Amoxicillin Clavulanate N = 3813 N (%)	Crude OR [95% CI], ARD	Adjusted OR [95% CI], ARD
Treatment failure—follow-up visits up to 30 days after the index date with ARS diagnosis and any antibiotic prescription	27 (4.9)	178 (4.7)	0.84 [0.76–1.45], −0.002	0.99 [0.66–1.52], −0.001
Chronic or Subacute rhinosinusitis diagnosis up to 90 days following the index date	3 (0.5)	97 (2.5)	4.80 [1.52–15.21], 0.020	4.62 [1.45–14.69], 0.019
Rhinosinusitis complication—A diagnosis of a complication of ARS within 90 days of the index date. Complications include orbital or periorbital cellulitis, osteomyelitis, meningitis, sinus vein thrombosis, septic cavernous sinus thrombosis, subperiosteal abscess, and intracranial abscess	6 (1.1)	44 (1.2)	1.06 [0.45–2.52], 0.0008	1.05 [0.44–2.52], 0.0004
Emergency room referral—A referral given on a visit with ARS diagnosis or one of the above complications within 90 days of the index date	6 (1.1)	44 (1.2)	1.07 [0.45–2.52], 0.0008	1.05 [0.44–2.52], 0.0004

## Data Availability

The original contributions presented in this study are included in this article. Further inquiries can be directed to the corresponding author.
